# Distinct Clinical Patterns Associated with HLA-DRB1 Genotypes in Pediatric Autoimmune Liver Diseases

**DOI:** 10.3390/ijms27209002

**Published:** 2026-10-11

**Authors:** Alina Grama, Alexandra Mititelu, Gabriel Benţa, Raisa Sucaciu, Daniel Popescu, Bianca Mariş, Alexia Onaciu, Ştefan Agoston, Tudor Lucian Pop

**Affiliations:** 1 2nd Pediatric Discipline, Department of Mother and Child, Iuliu Hatieganu University of Medicine and Pharmacy, 400012 Cluj-Napoca, Romania; alina.grama@umfcluj.ro (A.G.); sucaciu.raisamaria@elearn.umfcluj.ro (R.S.); daniel_popescu02@yahoo.com (D.P.); mateescu_bianca_raluca@elearn.umfcluj.ro (B.M.); onaciu.alexia@elearn.umfcluj.ro (A.O.); agoston.stefan@eleran.umfcluj.ro (Ş.A.); tudor.pop@umfcluj.ro (T.L.P.); 2 2nd Pediatric Clinic, Center of Expertise in Pediatric Liver Rare Diseases, Emergency Clinical Hospital for Children, 400177 Cluj-Napoca, Romania

**Keywords:** autoimmune hepatitis, autoimmune sclerosing cholangitis, pediatric autoimmune liver disease, HLA-DRB1, genotype–phenotype correlation, liver fibrosis

## Abstract

Autoimmune liver diseases (AILD), including autoimmune hepatitis (AIH) and autoimmune sclerosing cholangitis (ASC), are rare but important causes of chronic liver disease in children. Although HLA-DRB1 alleles have been associated with disease susceptibility, their role in modulating the clinical phenotype and long-term outcome of pediatric AILDs remains incompletely understood. We aimed to characterize the distribution of HLA-DRB1 alleles and genotype combinations and describe the accompanying clinical features in a pediatric AILD cohort. Thirty children diagnosed with AIH-1 (n = 14), AIH-2 (n = 6), or ASC (n = 10) underwent HLA-DRB1 genotyping. Clinical presentation, fibrosis stage, treatment response, and associated autoimmune diseases were retrospectively analyzed. The most frequent alleles copies were HLA-DRB1*03 (n = 15), predominantly identified in AIH, and HLA-DRB1*13 (n = 14), identified in both AIH and ASC. Genotype combinations involving these alleles were observed across a broad spectrum of clinical presentations, fibrosis stages, treatment responses, and extrahepatic autoimmune manifestations. In this small cohort, HLA-DRB1*14-containing genotypes were observed in several patients with severe disease features, including advanced fibrosis, cirrhosis, treatment-refractory disease, and ulcerative colitis. Patients carrying HLA-DRB1*11, HLA-DRB1*15, or HLA-DRB1*16 had favorable hepatic outcomes despite severe initial presentation or associated extrahepatic autoimmune manifestations; however, the number of carriers was small. Clinical features varied among patients carrying different genotype combinations; however, the independent contribution of the accompanying allele could not be assessed. Our findings illustrate marked clinical heterogeneity among children carrying different HLA-DRB1 alleles and genotype combinations. In this exploratory cohort, selected HLA-DRB1 combinations co-occurred with distinct clinical patterns. HLA profiling may have a role in future risk stratification and individualized follow-up of children with AILD, but these observations are hypothesis-generating, they do not support genotype-based clinical management and require validation in larger, multicenter cohorts.

## 1. Introduction

Autoimmune liver diseases (AILD) in childhood comprise a heterogeneous group of immune-mediated disorders, primarily including autoimmune hepatitis (AIH) and autoimmune sclerosing cholangitis (ASC). Although these conditions are relatively uncommon, they represent important causes of chronic liver disease in children and adolescents and may progress to advanced fibrosis, cirrhosis, and the need for liver transplantation (LT). Despite sharing several clinical, serological, and histopathological features, AIH and ASC differ in their immunological profiles, age at presentation, associated autoantibodies, and long-term clinical outcomes, suggesting distinct pathogenic mechanisms that underlie disease susceptibility and progression [1,2].

Genetic predisposition plays a central role in AILD. The human leukocyte antigen (HLA) region on chromosome 6 is the strongest susceptibility locus. HLA class II molecules regulate antigen presentation to CD4+ T lymphocytes, and HLA-DRB1 variation has consistently been associated with disease susceptibility, phenotype, and severity [3,4,5]. Several HLA-DRB1 alleles have been associated with specific AILD phenotypes. In Northern European and North American populations, HLA-DRB1*03 and HLA-DRB1*04 are considered the principal susceptibility alleles for AIH-1, and HLA-DRB1*07 and HLA-DRB1*14 for AIH-2 [5]. In pediatric studies, HLA-DRB1*13 was associated with ASC and overlap phenotypes, particularly in the context of inflammatory bowel disease (IBD) [3,5,6]. Compared with adults, HLA-DRB1*03- and HLA-DRB1*13-associated disease has been linked to more advanced fibrosis and a more severe phenotype at diagnosis [4]. Geographic and ethnic variability further supports population-specific HLA susceptibility patterns [6,7]. Despite these observations, most evidence derives from adult cohorts, whereas pediatric studies remain limited, particularly in Eastern Europe. Moreover, previous studies have primarily focused on individual HLA alleles rather than genotype combinations, leaving their contribution to phenotypic heterogeneity largely unexplored [3,5].

This study aims to describe HLA-DRB1 genotype combinations and associated clinical characteristics, fibrosis, treatment response, and extrahepatic autoimmunity in children with AILD, and to explore their potential role in disease stratification and personalized clinical management.

## 2. Results

### 2.1. Patient Characteristics

**Figure 1:**
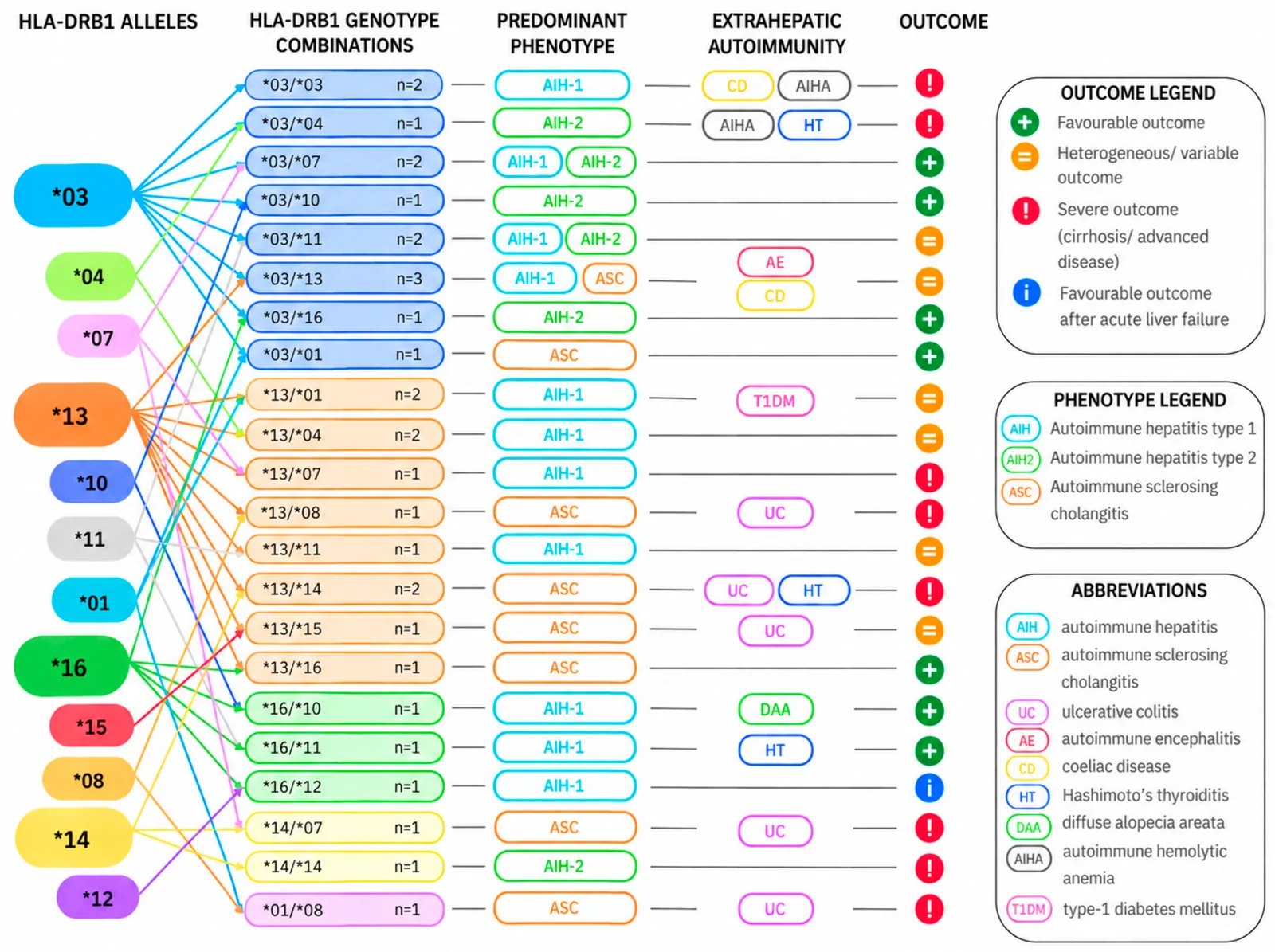
Clinical phenotype associated with HLA-DRB1 genotype combinations in the study cohort. (n represents the number of patients with each genotype combination).

The study included 30 children with AILD (median age 9.5 years; range: 1–17 years), comprising 22 girls (73.3%) and 8 boys (26.7%). Fourteen patients (46.7%) had AIH-1, six (20.0%) AIH-2, and ten (33.3%) ASC (Table 1). Associated autoimmune diseases were present in 13 patients (43.3%), whereas four (13.3%) had a positive family history of autoimmune disorders. Extrahepatic autoimmune manifestations included IBD, coeliac disease (CD), autoimmune endocrinopathies, autoimmune hemolytic anemia (AIHA), autoimmune encephalitis, alopecia areata, and cryoglobulinaemic vasculitis (Figure 1).

Clinical presentations ranged from mild disease to ALF or chronic liver disease, including cirrhosis. At diagnosis, fibrosis ranged from absent (F0) or minimal (F1) to moderate (F2), advanced (F3), or cirrhosis (F4). Four patients (13.3%) with advanced fibrosis or cirrhosis presented with portal hypertension (Table 1).

### 2.2. Distribution of HLA Alleles

HLA-DRB1*03 was the most frequently identified allele (25%), followed by HLA-DRB1*13 (23.3%). Compared with two Romanian reference populations [10,11], HLA-DRB1*03 and HLA-DRB1*13 were significantly more frequent in children with AILD (Table 2). HLA-DRB1*03 predominated in AIH, whereas HLA-DRB1*13 was identified in both AIH and ASC. HLA-DRB1*14 was observed primarily in patients with ASC and AIH-2 (Table 3).

## 3. Discussion

Previous studies, particularly the large multicentre cohort reported by Ma et al., have primarily established the role of individual HLA-DRB1 alleles in disease susceptibility [12]. Our study complements previous allele-level investigations by describing clinical heterogeneity among children carrying different HLA-DRB1 genotype combinations (fibrosis progression, treatment response, and extrahepatic autoimmunity in pediatric AILD). Clinical heterogeneity among patients carrying the same HLA-DRB1 allele suggests that disease expression may be influenced not only by individual susceptibility alleles but also by interactions within specific HLA-DRB1 genotypes.

HLA-DRB1 does not act in isolation. Associations attributed to DRB1 may reflect linkage disequilibrium with nearby class II loci, particularly HLA-DQA1 and HLA-DQB1, or with extended HLA haplotypes. In addition, HLA class I loci may contribute to disease susceptibility and phenotype through mechanisms distinct from class II-mediated CD4+ T-cell antigen presentation.

HLA-DRB1*01 has been reported as a susceptibility allele for AIH-1, together with HLA-DRB1*03 and HLA-DRB1*04, although its contribution appears to vary across different ethnic populations. While HLA-DRB1*01 has been associated with increased susceptibility in Japanese cohorts, the genetic risk profile observed in Northern European and North American populations is more commonly reported to be HLA-DRB1*03 and HLA-DRB1*04 [3,12]. In Japanese cohorts, HLA-DRB1*01 has been involved in the ASC–UC overlap phenotype.

In our study, HLA-DRB1*01 occurred in combination with HLA-DRB1*03, HLA-DRB1*08, and HLA-DRB1*13. The HLA-DRB1*01/*03 and HLA-DRB1*01/*08 genotypes were identified in patients with ASC, one of whom subsequently developed UC, consistent with previous reports. The HLA-DRB1*01/*13 genotype was identified in two patients with AIH-1 who presented with advanced fibrosis at diagnosis (F3 and F4, respectively). Both patients exhibited persistent p-ANCA positivity; however, neither showed radiological or histopathological features of cholangitis. Although no cholangiopathic phenotype became clinically apparent, the combination of advanced fibrosis and persistent p-ANCA positivity may indicate an underlying predisposition to cholangiopathy, warranting continued surveillance. They achieved sustained biochemical remission on immunosuppressive therapy, with no evidence of disease progression during follow-up. Longitudinal surveillance is warranted because ASC may become clinically apparent only during follow-up [2].

HLA-DRB1*03 and HLA-DRB1*04 are the principal HLA class II susceptibility alleles for AIH in populations of European ancestry. HLA-DRB1*03 has been associated with earlier onset and a more aggressive clinical course of the disease, whereas HLA-DRB1*04 is more frequently observed in patients presenting with later onset and milder clinical manifestations [3]. In our cohort, HLA-DRB1*03 was the most frequent allele (25%), present in 13 of 30 patients across both AIH and ASC phenotypes. It was identified across a broad spectrum of HLA-DRB1 genotype combinations: *03/*03, *03/*04, *03/*07, *03/*10, *03/*11, *03/*13, and *03/*16, reflecting considerable genetic heterogeneity. The two homozygous patients were both diagnosed with AIH-1 but had distinct clinical courses. One patient presented with ALF, cirrhosis, and AIHA requiring intensive immunosuppressive therapy. HLA-DRB1*03 has also been associated with several autoimmune diseases, including AIHA, autoimmune thyroid disorder and Sjögren’s syndrome [3,13]. The second had mild disease without fibrosis (F0) and developed CD during follow-up. The subsequent development of CD is consistent with the known association between HLA-DRB1*03 and the HLA-DQ2.5 haplotype [14]. The marked clinical variability observed among HLA-DRB1*03 carriers supports the concept that disease expression may be modified by the accompanying HLA allele. Similar findings have been reported in an Argentine cohort, with increased frequencies of HLA-DRB1*03 and HLA-DRB1*04 and lower frequencies of HLA-DRB1*08 and HLA-DRB1*11 in AIH [15]. A large European pediatric cohort further supported the role of HLA-DRB1*03, with homozygosity associated with increased susceptibility and a more aggressive phenotype [12]. HLA-DRB1*03 was more frequent in patients with AIH-1 than in those with ASC or AIH-2, particularly in its homozygous form [12]. Despite its association with more severe disease presentation, HLA-DRB1*03-positive patients achieved remission more rapidly than carriers of other HLA alleles, including HLA-DRB1*13, which was associated with a longer time to remission [12].

HLA-DRB1*03 has also been reported in pediatric ASC, although less frequently than HLA-DRB1*13 [12]. In our cohort, HLA-DRB1*03 was identified in three patients with ASC, in combination with HLA-DRB1*13 (n = 2) and HLA-DRB1*01 (n = 1). All presented with advanced liver fibrosis (F3–F4) at diagnosis. Sustained biochemical remission was achieved in all patients during follow-up, although one patient with the HLA-DRB1*03/*13 genotype experienced multiple disease relapses, necessitating escalation of immunosuppressive therapy.

HLA-DRB1*04 was identified in three patients, in combination with HLA-DRB1*03 or HLA-DRB1*13. Previous studies associated HLA-DRB1*04 with later disease onset and a milder clinical phenotype [12]; however, this pattern was not observed in our cohort. Two patients carrying the HLA-DRB1*04/*13 genotype presented with cirrhosis at diagnosis, although the concomitant presence of HLA-DRB1*13 precludes attributing disease severity to HLA-DRB1*04 alone. The third patient, carrying the HLA-DRB1*03/*04 genotype, presented with AIH-2, cirrhosis, portal hypertension, AIHA, and Hashimoto thyroiditis and achieved sustained clinical and biochemical remission during follow-up. Although HLA-DRB1*04 has been associated with susceptibility to systemic lupus erythematosus (SLE) [16,17], our patient did not fulfill the diagnostic criteria for SLE. Because of the small number of carriers and the coexistence of other susceptibility alleles, the independent contribution of HLA-DRB1*04 to disease phenotype could not be determined.

HLA-DRB1*07 is a well-established susceptibility allele for AIH-2, particularly in patients with anti-liver/kidney microsome type 1 (anti-LKM1) antibodies, and has been reported in association with HLA-DRB1*15, HLA-DRB1*04, and HLA-DQB1*06 [5,12]. In Spain, HLA-DRB1*07 has been strongly associated with AIH-2, with HLA-DQB1*02 occurring more frequently because of linkage disequilibrium between these loci [18]. In our cohort, HLA-DRB1*07 occurred in combination with HLA-DRB1*03, HLA-DRB1*13, and HLA-DRB1*14. The HLA-DRB1*07/*03 genotype was identified in two patients with AIH and distinct clinical phenotypes, including one infant with AIH-2, anti-LKM1 positivity, and ALF at presentation. Both achieved sustained biochemical remission following prednisone and azathioprine therapy. HLA-DRB1*07/*13 was identified in one patient with AIH-1 and cirrhosis, whereas HLA-DRB1*07/*14 occurred in one patient with ASC, UC, and advanced fibrosis.

HLA-DRB1*08 has been recognized as a susceptibility allele for AIH-1 in North Indian populations [19], whereas studies from Japan have suggested a potential protective role, with a significantly lower frequency among patients with AIH than healthy controls [20]. In our cohort, HLA-DRB1*08 occurred in combination with HLA-DRB1*13 and HLA-DRB1*01, two alleles previously implicated in susceptibility to the ASC–UC overlap phenotype [21]. Both genotype combinations were identified in children with early-onset ASC who subsequently developed UC and achieved sustained clinical remission without liver disease progression. These observations support the close immunogenetic relationship between ASC and UC and suggest that specific HLA-DRB1 genotype combinations may contribute to phenotypic heterogeneity in pediatric AILD.

HLA-DRB1*10 has not been recognized as a major susceptibility allele in AILD, and evidence regarding its clinical significance remains limited. In our cohort, HLA-DRB1*10 was identified in two patients with AIH-1 and AIH-2, in combination with HLA-DRB1*16 and HLA-DRB1*03, respectively. Both presented with relatively mild liver disease and favorable long-term hepatic outcomes. One patient developed diffuse alopecia areata during follow-up.

HLA-DRB1*11 has been proposed as a protective rather than a susceptibility allele in AIH-1, with lower frequencies reported in patients than in healthy controls [22]. In our cohort, HLA-DRB1*11 occurred in combination with HLA-DRB1*03, HLA-DRB1*13, and HLA-DRB1*16, exclusively in patients with AIH. The HLA-DRB1*03/*11 genotype was observed in two patients (AIH-1 and AIH-2), whereas HLA-DRB1*11/*13 and HLA-DRB1*11/*16 each occurred in one patient (AIH-1). Extrahepatic autoimmune manifestations included autoimmune encephalitis and Hashimoto thyroiditis. Consistent with its proposed protective role, all HLA-DRB1*11 carriers had favorable hepatic outcomes, with absent or minimal fibrosis and a good response to immunosuppressive therapy.

HLA-DRB1*13 is a major immunogenetic factor in AILD, although its clinical significance varies according to geographic region, age, and disease phenotype. It has been associated with increased susceptibility to AIH and ASC in several pediatric and South American cohorts but has also been reported to exert protective effects in other autoimmune diseases, including rheumatoid arthritis, SLE, and systemic sclerosis [23]. In populations where HLA-DRB1*03 and HLA-DRB1*04 are less prevalent, particularly in Argentina and Venezuela, HLA-DRB1*13 has emerged as an alternative susceptibility allele [5,24]. Similar population-specific associations have been reported in Turkish and Egyptian cohorts [25,26]. In our cohort, HLA-DRB1*13 was identified in 14 of 30 patients across both AIH and ASC. In AIH, it was most frequently associated with HLA-DRB1*01, HLA-DRB1*03, HLA-DRB1*04, and HLA-DRB1*07. Disease severity ranged from absent fibrosis to cirrhosis; however, most patients achieved favorable hepatic outcomes under immunosuppressive therapy. One patient carrying the HLA-DRB1*13/*01 genotype had pre-existing type 1 diabetes mellitus despite both alleles being considered protective in selected adult populations [27]. Persistent p-ANCA positivity was observed in patients carrying HLA-DRB1*13 in combination with HLA-DRB1*01, HLA-DRB1*04, or HLA-DRB1*11 despite the absence of radiological or histological evidence of ASC, suggesting a possible predisposition to biliary immune dysregulation. HLA-DRB1*13 has also been proposed to confer protection against CD; however, one patient with AIH-1 developed CD despite carrying HLA-DRB1*03, supporting the influence of accompanying HLA alleles on disease expression [28].

HLA-DRB1*13 is the HLA allele most consistently associated with pediatric ASC and with an increased risk of IBD, advanced fibrosis, and severe disease [10,29]. In our cohort, ASC was identified in seven HLA-DRB1*13-positive patients, four of whom had concomitant IBD. Clinical severity ranged from stable fibrosis to progressive cirrhosis. Two patients carrying the HLA-DRB1*13/*14 genotype showed distinct clinical courses, one progressing to cirrhosis despite prolonged therapy and recurrent IBD relapses, whereas the other remained clinically stable. UC was frequently observed in patients carrying HLA-DRB1*13 in combination with HLA-DRB1*03, HLA-DRB1*08, HLA-DRB1*14, HLA-DRB1*15, or HLA-DRB1*16, and one patient had persistently elevated fecal calprotectin without established IBD. Our observations support the reported increased risk of pediatric ASC associated with HLA-DRB1*13 and the extended DRB1*13:01-DQA1*01:03-DQB1*06:03 haplotype [29].

The involvement of HLA-DRB1*13 in both AIH-1 and ASC may reflect shared mechanisms of antigen presentation and T-cell activation [21]. As previously reported in pediatric cohorts, all patients in our cohort with concomitant ASC and UC carried HLA-DRB1*13 [30].

HLA-DRB1*14 has been associated with diverse clinical phenotypes across pediatric AILD. In Indian pediatric cohorts, it has been reported predominantly in AIH-2 and linked to elevated serum IgG levels, poorer response to immunosuppressive therapy, and more frequent disease relapse [19,28]. In our cohort, the only patient with a homozygous HLA-DRB1*14/*14 genotype had AIH-2 with cirrhosis at diagnosis. HLA-DRB1*14 also occurred in combination with HLA-DRB1*13, HLA-DRB1*07, and HLA-DRB1*03. One patient carrying the HLA-DRB1*13/*14 genotype had the most severe phenotype, characterized by cirrhosis, recurrent IBD flares, and autoimmune thyroiditis [32], whereas two patients carrying HLA-DRB1*14-containing genotypes subsequently developed UC. In Northern European cohorts, HLA-DRB1*14 has been reported to be protective in association with HLA-DRB1*15:01 [23,31]. These contrasting observations suggest that the clinical expression of HLA-DRB1*14 may be influenced by the accompanying HLA allele, particularly HLA-DRB1*03, HLA-DRB1*07, and HLA-DRB1*13.

HLA-DRB1*15 has generally been regarded as a protective allele against AIH and ASC in populations of Northern European ancestry [12]. Even when co-occurring with susceptibility alleles, it may modulate disease expression and contribute to a less aggressive hepatic phenotype [12,30]. In our cohort, HLA-DRB1*15 was identified in only one patient, in combination with HLA-DRB1*13. The patient had ASC and achieved complete regression of fibrosis after first-line immunosuppressive therapy but subsequently developed UC and cryoglobulinemic vasculitis. Although HLA-DRB1*15 has been associated with selected autoimmune vasculitic disorders, particularly PR3-ANCA-associated vasculitis, no direct association with cryoglobulinemic vasculitis has been reported [30]. This complex autoimmune phenotype likely reflects the broader immunogenetic background rather than the isolated effect of HLA-DRB1*15.

HLA-DRB1*16 has been linked to several autoantibody-mediated autoimmune disorders, including SLE, myasthenia gravis, neuromyelitis optica, and ANCA-associated vasculitis [33]. Although not considered a major susceptibility allele for AIH, HLA-DRB1*16 has been associated with AIH susceptibility in certain populations [29]. In contrast, Northern Italian studies have linked HLA-DRB1*16 haplotypes to an increased susceptibility to ASC [34]. In our cohort, HLA-DRB1*16 occurred in combination with HLA-DRB1*10, HLA-DRB1*11, and HLA-DRB1*12 in patients with AIH-1, with HLA-DRB1*03 in one patient with AIH-2, and with HLA-DRB1*13 in one patient with ASC. Despite ALF at presentation in three of the four patients with AIH, all HLA-DRB1*16 carriers achieved sustained biochemical remission and showed no evidence of fibrosis progression during follow-up.

### 3.1. Genotype–Phenotype Correlations of HLA-DRB1 Combinations

Analysis of HLA-DRB1 genotype combinations revealed distinct genotype–phenotype patterns across pediatric AILD. HLA-DRB1*03 was predominantly associated with AIH, whereas HLA-DRB1*13 was distributed across both AIH and ASC. HLA-DRB1*14-containing genotypes (*03/*14, *07/*14, *13/*14, and *14/*14) were observed in patients with severe phenotypes, including advanced fibrosis, cirrhosis, and ASC, in agreement with previous reports. In contrast, HLA-DRB1*11, HLA-DRB1*15, and HLA-DRB1*16 were generally associated with favorable hepatic outcomes. Despite ALF at presentation, HLA-DRB1*16 carriers achieved sustained biochemical remission without fibrosis progression, whereas the patient carrying HLA-DRB1*15 showed complete fibrosis regression despite subsequent extrahepatic autoimmune manifestations. Our observations suggest that the clinical phenotype of pediatric AILD is influenced not only by individual HLA-DRB1 alleles but also by specific genotype combinations within the broader immunogenetic background (Figure 1).

The frequent coexistence of UC among patients carrying HLA genotypes previously associated with cholangiopathic phenotypes reinforces the close relationship between hepatic and intestinal autoimmunity and suggests a shared immunogenetic basis. The broad spectrum of extrahepatic autoimmune disorders observed in our cohort indicates that pediatric AILD represents systemic immune dysregulation rather than an isolated organ-specific disease.

### 3.2. Limitations of the Study

Although our findings provide novel insights into genotype–phenotype correlations in pediatric AILD, they should be interpreted in light of several limitations. The retrospective, single-center design and small sample size, inherent to the rarity of pediatric AILD, limited statistical power and precluded robust multivariable analyses adjusted for potential confounders, including disease subtype, sex, age at diagnosis, treatment exposure, and duration of follow-up. Several HLA alleles and genotype combinations were represented by only one or a few patients; therefore, apparent phenotype–genotype patterns may reflect chance observations and should not be interpreted as evidence of causal or independent associations. The reference populations were drawn from published studies rather than from contemporaneously recruited controls and did not provide age-matched individual-level genotype data. We assessed only HLA-DRB1; we could not evaluate the contributions of other class II loci, class I loci, extended HLA haplotypes, or non-HLA genetic or environmental factors. Accordingly, our results should be regarded as descriptive and hypothesis-generating and require replication in larger prospective multicenter cohorts, ideally including harmonized phenotype definitions, standardized longitudinal fibrosis assessment, high-resolution extended HLA typing, and contemporaneously recruited appropriately matched controls.

## 4. Materials and Methods

### 4.1. Study Design and Population

This retrospective observational cohort study investigated genotype–phenotype associations among pediatric patients diagnosed with AILD and followed from March 2021 to March 2026.

Clinical data were collected retrospectively from medical records, and HLA-DRB1 genotyping was performed during routine follow-up. Eligible patients had a confirmed diagnosis of AIH-1, AIH-2, or ASC according to ESPGHAN/EASL criteria, available HLA-DRB1 genotyping, and adequate follow-up. Patients with incomplete medical records (n = 2) or loss to follow-up (n = 3) were excluded.

### 4.2. Diagnostic Evaluation

The diagnosis of AILD was established in accordance with the current ESPGHAN and EASL recommendations [1,2]. AIH diagnosis was based on a compatible clinical presentation, elevated serum aminotransferases, increased immunoglobulin G (IgG) concentrations, a characteristic autoantibody profile, exclusion of alternative causes of liver disease, and liver histology when available [1,2]. Liver biopsy was available in 27/30 patients. In the remaining three, whose parents declined biopsy, AIH-1 was diagnosed based on compatible clinical, biochemical, immunological, and serological findings together with the absence of cholangiographic abnormalities on magnetic resonance cholangiopancreatography (MRCP) [1,2]. Clinical presentation was classified as acute hepatitis, chronic liver disease, or acute liver failure (ALF). Extrahepatic autoimmune manifestations and family history of autoimmune disease were recorded.

### 4.3. Liver Fibrosis Assessment

Liver fibrosis was assessed using transient elastography (FibroScan^®^, Echosens, Paris, France), performed according to the manufacturer’s recommendations and interpreted using published pediatric data [8,9]. Transient elastography was performed at diagnosis, at 6 and 12 months, and annually thereafter as part of routine clinical follow-up. Liver stiffness measurements were interpreted using published pediatric cut-off values and correlated with histopathological findings when available [8,9]. Fibrosis was classified according to the METAVIR system (F0–F4). Serial transient elastography and, when available, histopathology were used to assess fibrosis progression or regression.

### 4.4. HLA Genotyping

HLA-DRB1 genotyping was performed in all patients at an accredited molecular genetics laboratory (Bioclinica Laboratories, Timișoara, Romania) using polymerase chain reaction with sequence-specific primers (PCR-SSP). HLA-DRB1 was selected because it is one of the best-established HLA loci associated with susceptibility to pediatric AIH and ASC. Other HLA class II loci, including HLA-DQA1 and HLA-DQB1, and HLA class I loci, including HLA-A, HLA-B, and HLA-C, were not systematically typed and were therefore not analyzed. HLA-DRB1 alleles and genotype combinations were analyzed in relation to disease phenotype, fibrosis stage, treatment response, and associated autoimmune manifestations.

### 4.5. Data Collection

Variables included age, sex, clinical presentation, disease subtype, fibrosis stage, cirrhosis, treatment response, associated autoimmune diseases, IBD, and family history of autoimmune disorders.

### 4.6. Statistical Analysis

Because of the study’s exploratory nature and small sample size, the statistical analysis was primarily descriptive. Continuous variables are presented as median and range or mean ± standard deviation, as appropriate, whereas categorical variables are presented as counts and percentages. HLA allele frequencies, genotype distributions, genotype combinations, and accompanying clinical characteristics were summarized descriptively. We did not perform inferential tests of genotype–phenotype associations or genotype-level comparisons with reference populations. For exploratory comparisons of HLA-DRB1 allele frequencies with published Romanian reference populations, allele counts were compared using two-sided Fisher’s exact tests. To limit overinterpretation from multiple exploratory allele comparisons, we present raw *p*-values alongside false-discovery-rate-adjusted *p*-values using the Benjamini–Hochberg procedure. Findings that are no longer significant after adjustment are interpreted cautiously.

### 4.7. Ethics

The study was conducted in accordance with the Declaration of Helsinki. The Ethics Committee of the Iuliu Hatieganu University of Medicine and Pharmacy, Cluj-Napoca, Romania, approved the retrospective analysis of anonymized clinical data and HLA-DRB1 genotyping data (protocol code AVZ89; approval date: 22 April 2026). Parents or legal guardians provided written informed consent for participation and/or genetic testing in accordance with institutional requirements.

## 5. Conclusions

This study highlights the marked immunogenetic heterogeneity underlying pediatric autoimmune liver diseases and demonstrates that individual HLA-DRB1 alleles alone cannot explain the clinical expression of AILD. While HLA-DRB1*03 was the principal susceptibility allele for AIH and HLA-DRB1*13 was associated with the broadest spectrum of clinical phenotypes, the accompanying HLA allele appeared to modulate disease phenotype, fibrosis severity, treatment response, and extrahepatic autoimmune manifestations.

In contrast to the generally favorable hepatic outcomes observed in carriers of HLA-DRB1*11, HLA-DRB1*15, and HLA-DRB1*16, HLA-DRB1*14 was associated with more severe disease manifestations, including advanced fibrosis, cirrhosis, treatment-refractory disease, and concomitant UC. The frequent coexistence of hepatic, intestinal, and systemic autoimmune disorders further supports a shared immunogenetic background linking these conditions.

Our findings suggest that HLA-DRB1 genotype combinations, rather than individual alleles, may influence disease phenotype and long-term outcome in pediatric AILD. These observations highlight the potential value of HLA profiling for future risk stratification, individualized follow-up, and a better understanding of disease heterogeneity. Larger multicenter studies using high-resolution typing of extended HLA class I and class II haplotypes are required to determine whether the observed patterns are reproducible and to define the independent contribution of HLA-DRB1.

## Figures and Tables

**Table 1:** Demographic and clinical characteristics of the pediatric AILD cohort.

Characteristics	Overall Cohort (n = 30)
Age at diagnosis, years	Median 9.5 (range 1–17)
Female sex; n (%)	22 (73.3)
Disease subtype; n (%)	
AIH-1	14 (46.6)
AIH-2	6 (20.0)
ASC	10 (33.3)
ALF at presentation; n (%)	7 (23.3)
Liver fibrosis stage at diagnosis (cut-off values) * [8,9]; n (%)	
F0—no fibrosis	11 (36.7)
F1—minimal fibrosis (<6.5 kPa)	3 (10.0)
F2—moderate fibrosis (6.5–8.0 kPa)	4 (13.3)
F3—advanced fibrosis (8.5–11.5 kPa)	5 (16.7)
F4—cirrhosis (>11.5 kPa)	7 (23.3)
Associated extrahepatic autoimmune manifestations; n (%)	13 (43.3)
IBD associated with ASC; n (%)	5 (16.7)
Positive family history of autoimmune diseases; n (%)	4 (13.3)

* No patient had a baseline liver stiffness measurement within the indeterminate interval (>8.0 to <8.5 kPa). Abbreviations: AIH, autoimmune hepatitis; ASC, autoimmune sclerosing cholangitis; ALF, acute liver failure; IBD, inflammatory bowel disease.

**Table 2:** Exploratory comparison of HLA-DRB1 allele frequencies in the pediatric AILD cohort with aggregate frequencies reported in two published Romanian reference populations (stem cell donor study and Transylvanian study) [10,11].

HLA-DRB1 Allele	Our Cohort AILD (n = 30) Allele Frequency (%)	Romanian Stem Cell Donors [10] (n = 297) Allele Frequency (%)	*p*-Raw	*p*-Adjusted	Transylvanian Study [11] (n = 2917) Allele Frequency (%)	*p*-Raw	*p*-Adjusted
DRB1*01	6.7	8.40	0.8077	1.0000	9.8	0.5174	0.7473
DRB1*02		0.25					
DRB1*03	25.0	12.10	**0.0088**	0.0617	11.4	**0.0032**	**0.0427**
DRB1*04	5.0	9.51	0.3457	0.6514	8.8	0.4864	0.7473
DRB1*05		0.12					
DRB1*07	6.7	9.26	0.6401	0.8962	10.6	0.4031	0.7473
DRB1*08	3.3				2.5	0.6631	0.8620
DRB1*09		0.12			0.2		
DRB1*10	3.3	1.73	0.3028	0.6514	1.1	0.1448	0.3765
DRB1*11	6.7	19.38	**0.0132**	0.0617	19.0	**0.0121**	0.0524
DRB1*12	1.7	1.23	0.5389	0.8383	1.7	1.0000	1.0000
DRB1*13	23.3	10.99	**0.0107**	0.0617	11.4	**0.0076**	**0.0495**
DRB1*14	8.3	5.31	0.3722	0.6514	4.3	0.1866	0.4043
DRB1*15	1.7	5.93	0.2390	0.6514	8.8	0.0607	0.1974
DRB1*16	8.3	13.95	0.3190	0.6514	10.3	0.8303	0.9812

Allele frequencies were compared using two-sided Fisher’s exact tests. *p*-values are exploratory (*p*-values < 0.05 are bold). Adjustment for multiple testing was performed separately for each comparison using the Benjamini–Hochberg false discovery rate method.

**Table 3:** Distribution of HLA-DRB1 alleles according to disease subtype and associated extrahepatic autoimmune manifestations, with previously reported associations.

HLA Allele (Allele Count)	AIH-1 (n = 14)	AIH-2 (n = 6)	ASC (n = 10)	Previously Reported HLA Association with AILD [5,6,7,12,13,14,15,16,17,18,19,20,21,22,23,24,25,26,27,28,29,30,31]
DRB1*01 (n = 4)	2	0	2	AIH susceptibility in a selected population
DRB1*03 (n = 15)	7	5	3	Major susceptibility allele for AIH-1
DRB1*04 (n = 3)	2	1	0	AIH-1 susceptibility allele
DRB1*07 (n = 4)	2	1	1	Frequently associated with AIH-2
DRB1*08 (n = 2)	0	0	2	Population-dependent association with AIH; uncertain role in ASC susceptibility
DRB1*10 (n = 2)	1	1	0	Limited evidence: clinical significance remains uncertain
DRB1*11 (n = 4)	3	1	0	Potential protective/modifying role in AIH
DRB1*12 (n = 1)	1	0	0	Unclear association
DRB1*13 (n = 14)	7	0	7	Well-established association with ASC; association with AIH reported in selected populations
DRB1*14 (n = 5)	0	2	3	Associated with AIH-2 and cholangiopathic phenotypes
DRB1*15 (n = 1)	0	0	1	Limited evidence; possible association with favorable outcome
DRB1*16 (n = 5)	3	1	1	Association with ASC reported in selected populations

Abbreviations: AILD, autoimmune liver disease; AIH, autoimmune hepatitis; ASC, autoimmune sclerosing cholangitis.

## Data Availability

The original contributions presented in this study are included in the article. Further inquiries can be directed to the corresponding authors.

## Abbreviations

The following abbreviations are used in this manuscript:

AIH	Autoimmune hepatitis
AILD	Autoimmune liver disease
AIHA	Autoimmune hemolytic anemia
ALF	Acute liver failure
ANCA	Antineutrophil cytoplasmic antibodies
ASC	Autoimmune sclerosing cholangitis
CD	Coeliac disease
EASL	European Association for the Study of the Liver
ESPGHAN	European Society for Paediatric Gastroenterology, Hepatology and Nutrition
HLA	Human leukocyte antigen
HLA-DRB1	Human leukocyte antigen DR beta 1
IBD	Inflammatory bowel disease
IgG	Immunoglobulin G
LT	Liver transplantation
METAVIR	Meta-analysis of Histological Data in Viral Hepatitis
MRCP	Magnetic resonance cholangiopancreatography
p-ANCA	Perinuclear antineutrophil cytoplasmic antibodies
PCR-SSP	Polymerase chain reaction with sequence-specific primers
PR3-ANCA	Proteinase 3 antineutrophil cytoplasmic antibodies
SLE	Systemic lupus erythematosus
UC	Ulcerative colitis
